# Apathy Associated with Alzheimer's Disease

**DOI:** 10.2174/0115672050350970241216072400

**Published:** 2024-12-23

**Authors:** Dan Wu, Bo Zhang, Yajuan Chang, Shuming Huang

**Affiliations:** 1 Institute of Chinese Medicine, Heilongjiang University of Chinese Medicine, Harbin, Heilongjiang, China;; 2 Department of Pathology, The First Affiliated Hospital of Heilongjiang University of Chinese Medicine, Harbin, Heilongjiang, China

**Keywords:** Alzheimer’s disease, apathy, neuropsychiatric, behaviorology, anterior cingulate cortex, therapeutic efficacy

## Abstract

**Introduction/Objective:**

Apathy is a multidimensional and complex disease that is the primary neuropsychiatric symptom among those diagnosed with Alzheimer's disease (AD). Yet, apathy in AD is sometimes underestimated.

**Methods:**

A systematic literature review was conducted using databases such as PubMed, Scopus, and Web of Science. The search utilized specific keywords related to apathy and Alzheimer's disease (*e.g.,* “apathy,” “Alzheimer's disease,” “neuropsychiatric symptoms,” “front-striatal circuitry”). The studies were selected based on pre-defined criteria, including publication date (within the last 10 years), peer-reviewed status, and relevance to neurobiological, neurochemical, and behavioral aspects of apathy in AD. The articles were screened through title and abstract reviews, followed by full-text evaluations to ensure they met the inclusion criteria, such as relevance to apathy in Alzheimer's patients, study design rigor, and methodological quality.

**Results:**

Some research on the behavioral and neurobiological characteristics of apathy in AD points to the role of the front-striatal circuitry, particularly the anterior cingulate cortex (ACC). In addition, we reviewed the neurochemical, neuropsychological, and neuropathological characteristics believed to be associated with apathy symptoms.

**Conclusion:**

The findings indicate that understanding the intricate neurobiological underpinnings of apathy in AD is crucial for developing targeted interventions. Our analysis suggests that a multimodal approach, incorporating both pharmacological and personalized non-pharmacological strategies, could enhance therapeutic efficacy and improve patient outcomes. This highlights the need for future research to explore these combined treatment modalities and their potential to alleviate apathy in AD patients.

## 1 INTRODUCTION

Alzheimer's disease (AD) is a progressive neurodegenerative disorder characterized by the accumulation of amyloid-beta plaques and tau tangles in the brain, leading to synaptic dysfunction, neuroinflammation, and neuronal loss [1, 2]. Clinically, AD manifests as a spectrum of cognitive impairments, primarily affecting memory, executive function, and language, along with neuropsychiatric symptoms such as apathy and depression [3]. It is the most prevalent cause of dementia in older adults, contributing to significant morbidity and mortality worldwide [4]. The pathology of AD reflects complex interactions between genetic, environmental, and lifestyle factors, making it a multifactorial disease rather than merely a cognitive impairment [5].

Apathy is considered the most common neuropsychiatric symptom of Alzheimer's disease, and it is also frequently undervalued, receiving relatively little attention in literature and clinical practice. Although estimates vary, at some point in the disease process, it may affect upto 70% of AD patients [6]. Reports suggest that about 32.1% to 93.2% of Alzheimer's disease patients experience apathy symptoms [7-9], and approximately 42% of those with mild cognitive impairment, 80% with moderate cognitive impairment, and 92% with severe mental impairment exhibit apathy symptoms 6. Apathy is a condition of lacking or declining motivation in at least two domains-behavior aimed at achieving goals, cognitive processes, or emotions-resulting in substantial impairment in everyday functioning [10]. Studies conducted on both healthy individuals and patients with apathy have confirmed a novel viewpoint that apathy is not a singular phenomenon but rather an intricate multifaceted disease, potentially encompassing several social and emotional impairments [11, 12]. Affective apathy is often defined as the absence of recognition, interest, attention, and emotional reaction to irrelevant external events, leading to a decrease in goal-directed behavior. Significantly, apathy in AD is a highly painful symptom [13] for caregivers [14-16], and it is linked to a decrease in quality of life [17], an increase in incidence 18, and an increasing incidence of the disease [18].

**Fig. (1):**
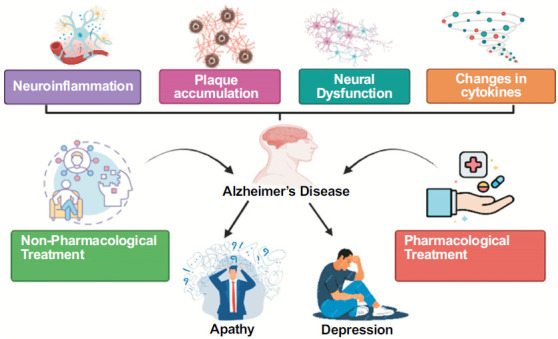
Association of AD with the progression of Apathy and depression and an overview of its treatment.

Although different from one another, apathy frequently coincides with depression (Fig. **1**). Although there are certain similarities between symptoms of apathy and depression, such as social disengagement or decreased involvement, pure apathy is distinct from symptoms prevalent in depression, such as regret, despair, or sadness [19-21]. There is emotional consistency in the emotional changes in depression (sadness, emptiness, guilt, suicidal thoughts), which is not typical of apathy [19, 21, 22]. In both apathy and depression, there seems to be an overlap in functional impairment of certain brain regions. For example, the involvement of some subcortical loops (such as front ostriatal loops) is associated with both apathy and depression [23-25]. On the other hand, recent evidence has shown that in diseases such as AD [21, 26], FTD [26], and PD [27, 28], apathy and depression differ in characteristics and lesion locations when they co-occur. The left orbitofrontal area exhibits reduced perfusion in cases of apathy, whereas the dorsolateral PFC region is linked to depression [8]. In addition, apart from the commonality of sadness, the coexistence of other neuropsychiatric symptoms, such as psychosis, agitation, or violence, can further complicate the detection and assessment of apathy by professionals in the field [29].

## 2 SYSTEMATIC LITERATURE REVIEW METHOD

A systematic literature review was conducted to gather and analyze the most recent research on apathy in Alzheimer's disease (AD). The methodology involved searching multiple databases, including PubMed, Scopus, and Web of Science, using specific keywords like “apathy,” “Alzheimer's disease,” “neuropsychiatric symptoms,” and “fronto-striatal circuitry.” Studies were selected based on their relevance, rigor, and recency to provide a comprehensive overview of the risk factors, behavioral and neurobiological features, and therapeutic approaches related to apathy in AD. This systematic approach ensures a thorough and unbiased synthesis of existing knowledge, allowing for a more accurate understanding of the disorder, guiding future research directions, and highlighting gaps in current therapeutic strategies.

## 3 RISK FACTORS FOR APATHY IN AD

Several studies have investigated the potential risk factors for apathy in AD [30]. A recent meta-analysis showed that apathy symptoms appear to be more severe in male patients [31], while several studies have emphasized the role of the potential severity of AD (such as longer duration of AD and worse cognitive abilities) [32, 33]. This is also consistent with the association of apathy severity with AD biomarkers, such as lower Aβ-42 and elevated p-tau [34, 35]. The relationship between apolipoprotein E and apathy or affective symptoms in AD patients is not yet fully determined and requires further research to validate [36]. The relationship between peripheral inflammatory markers such as TNF-α, IL-1, and IL-10 and apathy has been confirmed in many studies [37, 38]. In studies of AD patients' cerebrospinal fluid, changes in cytokine and cortisol levels have been found, that are linked with neuropsychiatric symptoms in patients of AD [39, 40]. This finding deserves further study for confirmation. Psychological studies of AD have emphasized that pre-disease personality, higher midlife motivational capacity [22], and increased basal levels of apathy [41] may serve as indicators of risk for apathy in people with AD. The etiology of apathy in AD is multifaceted, involving both pathophysiology and variable individual factors. Separating the specific risk factors based on fundamental disease processes is a challenging task, making this an area of potential future research interest.

## 4 BEHAVIOROLOGY FOR APATHY IN AD

Research on the behavioral characteristics of apathy in AD is very limited. In a study on how apathy in AD is related to decision-making, the authors did not categorize the AD patients into gradient groups based on the severity of symptoms, *i.e.,* they did not distinguish between the forgetful mild cognitive impairment group, the AD group, and the healthy control group, because there was no interaction between groups in terms of decision-making patterns. Therefore, apathy might have a general impact on reward-based decision-making, which is not necessarily specific to AD pathology [42]. A further study that included individuals with apathy-type AD and apathy-type Parkinson's disease-related dementia (PDD) revealed significant variations in executive function across the two cohorts. Apathetic patients exhibited impairments in several domains, including semantic fluency, motor response inhibition, and abstract thinking, when compared to non-dementia patients [43]. This study suggests that executive impairments related to apathy may be mediated by the prefrontal cortex. Additionally, due to the similarities between apathy-type AD and apathy-type PDD patients, the authors considered it a universal syndrome of behavioral and cognitive executive impairment, which is the basis for apathy in neurodegenerative diseases. Another study assessed attention biases in apathetic AD [44]. The application of eye-tracking technology revealed that individuals with apathetic AD allocated less time to social activities, but they could not draw clear conclusions about the relationship between apathy and any subdomain.

## 5 NEUROBIOLOGY FOR APATHY IN AD

### 5.1 The Potential Neurobiological Network for Apathy in AD Patients

**Fig. (2):**
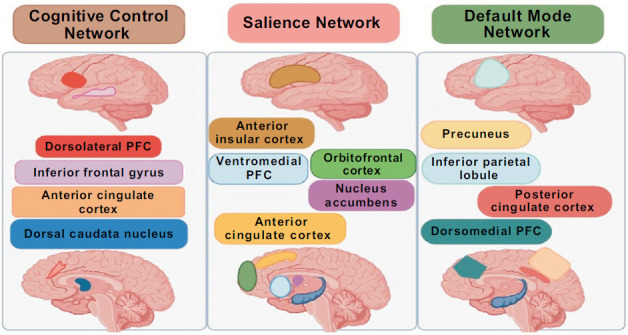
Three key neutral networks involved in goal-directed behavior and planning that are affected by apathy in AD.

Apathy is a clinical syndrome that may be caused by functional connection defects between discontinuous cortical and subcortical brain regions [45, 46]. These impaired connections affect both the primary functions of the neuroanatomical region and the network functions of these related cortical and subcortical areas (Fig. **2**) [47].

The Cognitive Control Network (CCN), also known as the Execution Control Network [48], includes key regions such as the Dorsolateral Prefrontal Cortex (DLPFC), Inferior Frontal Gyrus (IFG), Caudate Nucleus, and Dorsal Anterior Cingulate Cortex (ACC) [49]. Research suggests that reduced metabolic function and functional connectivity in these regions can lead to a decrease in goal-directed behaviors. In particular, this leads to deficits in the processes of preparing, rule discoveries, and set-shifting, which are crucial for the development, maintenance, and control of objectives [50].

The Ventral Attention Network, sometimes referred to as the Salience Network (SN), comprises critical brain regions, including the Orbitofrontal Cortex (OFC), Ventromedial Prefrontal Cortex (VMPFC), Anterior Cingulate Cortex (ACC), Amygdala, Ventral Striatum (containing the Nucleus Accumbens), and Anterior Insula [51]. A range of behavioral activities, including valuation (including the intrinsic value or value assessment of goods or behaviors) [52], social process systems, assessment of negative or prolonged threats, and the sensation of loss or frustrating non-reward, are all influenced by the SN. Neuronal activity in the (OFC) and VMPFC is strongly correlated with the significance of task events, encompassing the assessment of external environmental variables and internally relevant information of the individual [53]. The link between lesions in the OFC-VMPFC and apathy can be attributed to the observation that dysfunction in these regions can result in emotional blunting [54, 55]. The anterior insula has a role in the cerebral processing of emotionally important stimuli, including the regulation of sensorimotor states and interpersonal emotions [56], and specific neurons in the ACC are associated with rapid, intuitive assessments of certain complex situations, such as human social networks [57]. In summary, metabolic reductions or functional connectivity losses in these regions may lead to functional impairments in perceiving social or emotional value.

A related brain network with self-cognition is the Default Mode Network (DMN), which includes areas such as the Dorsomedial Prefrontal Cortex (DMPFC), Posterior Cingulate Cortex (PCC), Precuneus, Hippocampus, and Inferior Parietal Lobe [58]. The Precuneus is implicated in the process of self-awareness [59], while loops, including the DMPFC, exert a pivotal influence on the theory of mind [60]. Reduced neuronal activity in the frontal dorsomedial area results in impaired initiation and diminished energy levels. Moreover, malfunction in these areas might lead to patients experiencing challenges in self-motivation in terms of cognition or conduct, absence of spontaneous emotional reactions, and deficiency of self-generated ideas.

### 5.2 The Potential Neurochemical Basis of Apathy in AD Patients

**Fig. (3):**
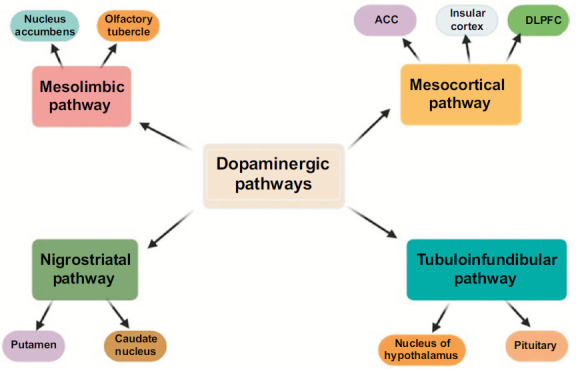
The four primary central dopaminergic modulatory pathways.

The catecholamines dopamine and norepinephrine (NA) are both produced from tyrosine. Dopamine is transformed into NA by the enzyme dopamine β-hydroxylase. The neurotransmitter dopamine plays a crucial role in facilitating good decision-making, and any impairment in its functioning is often linked to the clinical symptoms of apathy. Dopamine confers importance to reward stimuli and helps to manage the costs associated with exertion [61, 62]. Dopaminergic neurons provide innervation to many striatal circuits that regulate reward response (Fig. **3**) [63]. In cases of apathy-related AD, the dopamine indices in the putamen are decreased [64], and the subjective reward is diminished after the administration of the dopaminergic medication dextroamphetamine [65].

The locus coeruleus (LC) releases central NA, a crucial neurotransmitter implicated in processing rewards and cognition. Imaging the LC, which is situated in the brainstem, is challenging. Extensive subcortical and cortical projections emanate from the locus coeruleus norepinephrine (LC-NA) structure. LC neurons display a dual discharge pattern, with one pattern indicating the established phase and the other pattern indicating experimental tension [66, 67]. Previous research has demonstrated that impairment of the lateral cortex or dopaminergic neurons might impact the functioning of each other's systems [68, 69]. Additionally, the deterioration of the lateral cortex happens at an earlier stage and with greater severity in Parkinson's disease (PD) and AD compared to other brainstem nuclei or basal ganglia [70]. Therefore, it is suggested that an inability to LC-NA control is a primary factor contributing to early cognitive impairment and is linked to the early advancement of the disease [71, 72].

### 5.3 Neuropsychology of Apathy in AD Patients

Previous research has demonstrated that individuals with AD exhibit poor efficiency in managing many activities [73]. Furthermore, =a notable association between the executive role and both the frequency of errors produced during task execution and the level of apathy in AD still exists [74]. Apathy linked to moderate cognitive impairment (MCI) and AD commonly presents with early executive function impairments characterized by poor initiation. However, in the later stages of AD, these specific connections are often concealed by severe neuropathological indications [75]. The findings of a 2023 study on individuals with apathetic dementia revealed a correlation between social reward and emotional apathy [76], indicating impairments in reward-driven decision-making within the apathy syndrome.

### 5.4 Neuropathology of Apathy in AD Patients

Recently, studies have graded AD patients based on the degree of cognitive decline [77]. Research has demonstrated that apathy can manifest at any point during the evolution of cognitive function in AD and dementia. Moreover, the pathogenic changes in AD are associated with cognitive impairments and possibly neuropsychiatric disabilities. Adhering to this perspective, there have been reported instances of variations in brain architecture among individuals diagnosed with apathetic AD. Evidence suggests that there is an elevated accumulation of Aβ in the cortical areas of individuals with MCI [78], as well as in both bilateral frontal lobes and the right anterior cingulate gyrus of patients with AD [79, 80]. An analysis of the Apathy Evaluation Scale (AES) revealed that an elevation in apathy symptoms was linked to an increase in total Aβ deposition, while no such relationship was observed with depression symptoms [81]. Further studies have indicated that Aβ, independent of cognitive function, mild to moderately influences the development of apathy over time [82].

## 6 TREATMENT OF APATHY IN AD PATIENTS

### 6.1 Pharmacological Treatment of Apathy in AD Patients

Currently, the standard treatment for apathy in AD is cholinesterase inhibitors (ChIs), such as donepezil, which can increase the levels and duration of action of acetylcholine. AD, along with apathy-related AD, is associated with cholinergic dysfunction caused by disconnection of the cholinergic pathway. ChIs can moderately improve the overall cognitive function of AD patients [83], however, they have not yet been proven to be effective in the long-term treatment of apathy in AD. A clinical trial compared the combined treatment of donepezil and the cholinergic precursor phosphate choline (N=56) with the donepezil alone group (N=57). The results showed that the combined treatment group had significantly lower apathy scores. Unrelated to total cognitive performance, these findings were associated with executive function at baseline, indicating that individuals with lower apathy and preserved more effective executive function appeared to derive the greatest benefit from combined therapy [84].

Methylphenidate is a catecholamine/dopamine enhancer that can improve norepinephrine and dopamine activity in the striatal pathway associated with AD. Trials have evaluated the efficacy of methylphenidate on apathy related to AD [85, 86], proving that methylphenidate can improve the Apathy Evaluation Scale (AES) [87]. Furthermore, a separate study revealed that methylphenidate has a positive impact on apathy as assessed by the Neuropsychiatric Inventory (NPI) but not by the AES [88].

There are different methods of evaluating apathy in AD, these include observation of patient behaviors and self-reports from caregivers. The Apathy Evaluation Scale (AES) and Neuropsychiatric Inventory (NPI) are two questionnaires used to assess the caregivers’ reports on the motivation and interest of the patient. These assessments are useful in relating the amount of apathy as they take into consideration observations from both the clinical and personal views (Table **1**).

Additionally, although modafinil and atomoxetine have both been tested in the treatment of apathy related to AD, there is currently no evidence of clinical benefits. ChIs appear to be the most efficacious. The potential enhancement of these effects by the supplementary usage of cholinergic precursors or methylphenidate has yet to be confirmed.

### 6.2 Non-Pharmacological Treatments for Apathy in AD Patients

**Fig. (4):**
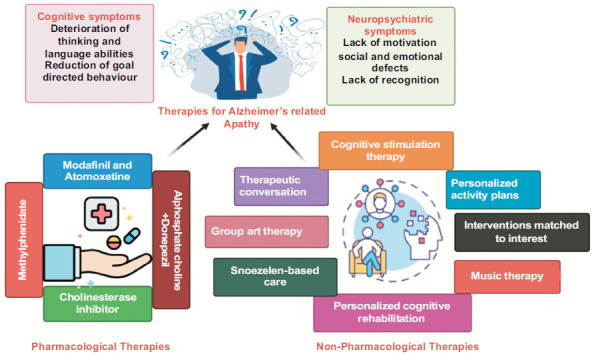
Pharmacological and non-pharmacological therapies for Apathy in AD patients.

Several non-pharmacological treatments, such as music-based programs, have demonstrated favorable outcomes in reducing apathy in senior individuals with dementia [89, 90]. Systematic individual interaction customized to the unique abilities or interests of the patient has also enhanced the reduction of apathy and other neuropsychiatric problems in individuals diagnosed with dementia [91, 92]. A recent evaluation of non-pharmacological therapies for apathy in AD indicates that interventions customized to individual interests or personalized activity plans, personalized cognitive rehabilitation, multimodal behavior or music therapy, and cognitive stimulation treatment may result in certain enhancements [93]. Furthermore, studies have demonstrated that therapeutic discussion [94], group art therapy [95], and Snoezelen-based care, which involve controlled multisensory environments, can effectively decrease apathy in dementia patients [96] (Fig. **4**). The integration of music, art, psychomotor skills, and pantomime has consistently decreased apathy in groups of individuals diagnosed with dementia. Exploring the combination of specific non-drug training in future studies for the treatment of apathy in AD may be a crucial line of inquiry.

## 7 PRECLINICAL STUDIES INDICATING APATHY IN ALZHEIMER'S DISEASE MODELS

Preclinical studies shown in Table **2** utilizing various animal models of Alzheimer's disease (AD) elucidated the underlying mechanisms and molecular players involved in the development of apathy. These studies demonstrate that alterations in brain circuitry, particularly within the frontostriatal pathways, are critical in mediating motivational deficits. Additionally, specific molecular factors, including neurotransmitters and neurotrophic proteins, have been identified as key players in the neurobiological processes contributing to apathy in AD.

## 8 DISCUSSION OF NEUROBIOLOGICAL AND BEHAVIORAL RESULTS ON APATHY IN ALZHEIMER'S DISEASE

The results of our analysis underscore the pivotal role of frontostriatal circuitry in the manifestation of apathy among individuals with Alzheimer's disease. Specifically, findings indicate that dysfunction within the anterior cingulate cortex (ACC) may contribute significantly to the behavioral and emotional disturbances characteristic of apathy. Neuroimaging studies reveal altered activation patterns in the ACC, correlating with reduced motivation and engagement in daily activities. Furthermore, neurochemical assessments suggest that imbalances in neurotransmitters, particularly dopamine and serotonin, may exacerbate apathy symptoms. The neuropsychological profile associated with apathy in AD highlights impairments in executive function, emotional regulation, and social cognition, which collectively hinder patients' ability to initiate and sustain goal-directed behavior. These insights not only enhance our understanding of the underlying mechanisms of apathy in AD but also emphasize the need for targeted interventions that address both the neurobiological and behavioral components of this debilitating symptom. Additionally, the identification of specific risk factors, such as age and comorbidities, may help clinicians better predict the onset of apathy in patients with AD. This knowledge can guide the development of personalized treatment plans, ultimately enchancing patient outcomes and quality of life. Future research should aim to explore the efficacy of novel therapeutic approaches that specifically target the front striatal circuitry and its associated neurochemical pathways.

## CONCLUSION

In this review, we assessed recent research on apathy in AD, focusing on risk factors, behavioral and neurobiological studies, and treatment approaches. There is a consensus that damage to the striatal circuit, particularly the subcortical connections between the ACC and OFC, is a key pathological basis for the manifestation of apathy in AD and is associated with the pathological characteristics of AD in these regions.

However, our understanding of the cognitive and behavioral mechanisms of apathy in AD patients is still limited. The most common treatment for apathy in AD is ChIs, but personalized non-pharmacological treatments have also provided some promising results. While apathy is now acknowledged as the predominant neuropsychiatric symptom of AD, the specific behavioral and neurobiological features of the apathy syndrome in AD have yet to be fully characterized. This is of greatest significance for the advancement of more effective therapy strategies.

## Figures and Tables

**Table 1:** Assessment tools for apathy in AD by different aspects of patients and caregiver insights.

**Sr. No.**	**Assessment Tool**	**Description**	**Strengths**	**Limitations**	**References**
1	Apathy Evaluation Scale (AES)	Measures apathy through clinician, patient, and informant ratings	Comprehensive; multi-perspective	Time-consuming; requires training	(Mast *et al.*, 2022; Radakovic *et al.*, 2020)
2	Neuropsychiatric Inventory (NPI)	Assesses behavioral disturbances in dementia, including apathy, *via* caregiver interviews	Widely used; captures broad symptoms	May not fully capture apathy; caregiver bias possible	(Lekhutlile, 2021; Tumati *et al.*, 2019)
3	Apathy Inventory (AI)	Evaluates emotional, cognitive, and behavioral apathy through caregiver and clinician ratings	Brief; focused on apathy	Limited scope	(Agüera-Ortiz *et al.*, 2015; M. Mortby *et al.*, 2021)
4	Dimensional Apathy Scale (DAS)	Measures executive, emotional, and initiation apathy *via* patient and informant reports	Differentiates apathy types; valid across conditions	Complex scoring; relies on informant details	(Radakovic *et al.*, 2020; Radakovic *et al.*, 2016)
5	Apathy-Motivation Index (AMI)	Self-report scale on motivation in various domains	Simple; patient perspective	Self-report bias; depends on patient's cognitive ability	(Altieri *et al.*, 2023; Klar *et al.*, 2022)
6	Lille Apathy Rating Scale (LARS)	Structured interview assessing nine apathy domains	Comprehensive; validated for neurodegenerative conditions	Lengthy; needs trained administration	(Fernández-Matarrubia *et al.*, 2016; Leśniak *et al.*, 2022)
7	Frontal Systems Behavior Scale (FrSBe)	Measures apathy, disinhibition, and executive dysfunction through self and informant ratings	Covers frontal lobe function; validated	Lengthy; needs both self and informant reports	(Cabrera *et al.*, 2016; RANGE & Practice, 2023)
8	Apathy Scale (AS)	Self-report questionnaire derived from AES, focusing on general apathy symptoms	Quick; useful for screening	Limited depth	(Calamia *et al.*, 2023; Ilardi *et al.*, 2024)
9	Initiative and Social Participation (ISP) Scale	Measures initiative and social participation as apathy indicators	Focuses on social aspects; relevant for social withdrawal	May not address non-social apathy; limited validation	(Andersson, 2000; Ang *et al.*, 2017)

**Table 2:** Summary of preclinical studies investigating apathy in Alzheimer's disease models and associated mechanisms.

**Model**	**Findings**	**Associated Mechanisms**	**Molecular Players**	**References**
APP/PS1 Transgenic Mice	Observed reduced exploration and motivation behaviors	Impaired frontostriatal circuitry	BDNF, Dopamine	[97]
Tau Transgenic Mice	Apathy-like behavior correlated with cognitive decline	Altered ACC activity	Serotonin, Neurotrophic factors	[98]
5xFAD Mice	Reduced engagement in rewarding tasks	Neuroinflammation affecting motivation	IL-1β, TNF-α	[99]
Double Transgenic (APP/TAU) Mice	Increased apathy in later stages of AD	Disruption of dopaminergic signaling	Dopamine receptors, Glutamate	[100]
Knockout Models (BDNF)	Significant anhedonia and lack of initiative	Dysregulation of motivational pathways	BDNF, ERK signaling pathway	[101]
APP/PS1 Mice	Impaired decision-making and motivation	Altered frontal cortex function	Dopamine, Norepinephrine	[102]
Tau Mice	Increased passive behavior and social withdrawal	Impairment of the reward system	Serotonin, Dopaminergic pathways	[103]
Tg2576 Mice	Reduced interest in novel environments	Changes in neurotransmitter levels	BDNF, Dopamine, Serotonin	[104]
5xFAD Mice	Apathy-like behavior linked to neuroinflammation	Inflammatory cytokine activity	IL-6, TNF-α	[105]
APP/PS1 and Tau Mice	Behavioral deficits correlated with neuroanatomical changes	Disruption in neural connectivity	Neurotrophic factors, Amyloid-beta	[106]

## LIST OF ABBREVIATIONS

DLPFC: Dorsolateral Prefrontal Cortex
IFG: Inferior Frontal Gyrus
CCN: Cognitive Control Network
PDD: Parkinson’s Disease-related Dementia
ACC: Anterior Cingulate Cortex
AD: Alzheimer’s Disease

## References

[1] Tahir M., Kang M.H., Park T.J. (2024). Multifaceted neuroprotective approach of Trolox in Alzheimer’s disease mouse model: Targeting Aβ pathology, neuroinflammation, oxidative stress, and synaptic dysfunction.. Front. Cell. Neurosci..

[2] Iliyasu M.O., Musa S.A., Oladele S.B., Iliya A.I. (2023). Amyloid-beta aggregation implicates multiple pathways in Alzheimer’s disease: Understanding the mechanisms.. Front. Neurosci..

[3] Mougias M., Beratis I.N., Moustaka K., Alexopoulos P., Assimakopoulos K. (2023). The differential role of executive apathy in Alzheimer’s disease dementia, mild cognitive impairment and healthy cognitive ageing.. Geriatrics.

[4] Lanctôt K.L., Hahn-Pedersen J.H., Eichinger C. (2024). Burden of illness in people with Alzheimer’s disease: A systematic review of epidemiology, comorbidities and mortality.. J. Prev. Alzheimers Dis..

[5] Ourry V., Binette A.P., St-Onge F. (2024). How do modifiable risk factors affect Alzheimer’s disease pathology or mitigate its effect on clinical symptom expression?. Biol. Psychiatry.

[6] Mega M.S., Cummings J.L., Fiorello T., Gornbein J. (1996). The spectrum of behavioral changes in Alzheimer’s disease.. Neurology.

[7] van Reekum R., Stuss D.T., Ostrander L. (2005). Apathy: Why care?. J. Neuropsychiatry Clin. Neurosci..

[8] Holthoff V.A., Beuthien-Baumann B., Kalbe E. (2005). Regional cerebral metabolism in early Alzheimer’s disease with clinically significant apathy or depression.. Biol. Psychiatry.

[9] Srikanth S., Nagaraja A.V., Ratnavalli E. (2005). Neuropsychiatric symptoms in dementia-frequency, relationship to dementia severity and comparison in Alzheimer’s disease, vascular dementia and frontotemporal dementia.. J. Neurol. Sci..

[10] Robert P.H., Mulin E., Malléa P., David R. (2010). Review: Apathy diagnosis, assessment, and treatment in Alzheimer’s disease.. CNS Neurosci. Ther..

[11] Ang Y.S., Lockwood P., Apps M.A.J., Muhammed K., Husain M. (2017). Distinct subtypes of apathy revealed by the apathy motivation index.. PLoS One.

[12] Radakovic R., Starr J.M., Abrahams S. (2017). A novel assessment and profiling of multidimensional apathy in Alzheimer’s disease.. J. Alzheimers Dis..

[13] Dolphin H., Dyer A.H., McHale C., O’Dowd S., Kennelly S.P. (2023). An update on apathy in Alzheimer’s disease.. Geriatrics.

[14] Dauphinot V., Delphin-Combe F., Mouchoux C. (2015). Risk factors of caregiver burden among patients with Alzheimer’s disease or related disorders: A cross-sectional study.. J. Alzheimers Dis..

[15] Riedijk D.V.M., Duivenvoorden H.J., Niermeijer M.F., Van Swieten J.C. (2006). Caregiver burden, healthrelated quality of life and coping in dementia caregivers: A comparison of frontotemporal dementia and Alzheimer’s disease.. Dement. Geriatr. Cogn. Disord..

[16] Chen CT, Chang CC, Chang WN (2017). Neuropsychiatric symptoms in Alzheimer’s disease: Associations with caregiver burden and treatment outcomes.

[17] Hongisto K, Hallikainen I, Selander T (2017). Quality of life in relation to neuropsychiatric symptoms in Alzheimer’s disease: 5-year prospective ALSOVA cohort study.. Int J Geriatr Psychiatry.

[18] Spalletta G., Long J.D., Robinson R.G. (2015). Longitudinal neuropsychiatric predictors of death in Alzheimer’s disease.. J. Alzheimers Dis..

[19] Benoit M., Berrut G., Doussaint J. (2012). Apathy and depression in mild Alzheimer’s disease: A cross-sectional study using diagnostic criteria.. J. Alzheimers Dis..

[20] Benoit M., Andrieu S., Lechowski L., Gillette-Guyonnet S., Robert P.H., Vellas B. (2008). Apathy and depression in Alzheimer’s disease are associated with functional deficit and psychotropic prescription.. Int. J. Geriatr. Psychiatry.

[21] Starkstein S.E., Ingram L., Garau M.L., Mizrahi R. (2005). On the overlap between apathy and depression in dementia.. J. Neurol. Neurosurg. Psychiatry.

[22] Mortby M.E., Maercker A., Forstmeier S. (2011). Midlife motivational abilities predict apathy and depression in Alzheimer disease: The aging, demographics, and memory study.. J. Geriatr. Psychiatry Neurol..

[23] Hirono N., Mori E., Ishii K. (1998). Frontal lobe hypometabolism and depression in Alzheimer’s disease.. Neurology.

[24] Landes A.M., Sperry S.D., Strauss M.E., Geldmacher D.S. (2001). Apathy in Alzheimer’s disease.. J. Am. Geriatr. Soc..

[25] Lopez O.L., Živković S., Smith G., Becker J.T., Meltzer C.C., DeKosky S.T. (2001). Psychiatric symptoms associated with cortical-subcortical dysfunction in Alzheimer’s disease.. J. Neuropsychiatry Clin. Neurosci..

[26] Basavaraju R., Feng X., France J., Huey E.D., Provenzano F.A. (2022). Depression is associated with preserved cortical thickness relative to apathy in frontotemporal dementia.. J. Geriatr. Psychiatry Neurol..

[27] Camargo C.H.F., Serpa R.A., Jobbins V.A., Berbetz F.A., Sabatini J.S. (2018). Differentiating between apathy and depression in patients with parkinson disease dementia.. Am. J. Alzheimers Dis. Other Demen..

[28] Kirsch-Darrow L., Marsiske M., Okun M.S., Bauer R., Bowers D. (2011). Apathy and depression: Separate factors in parkinson’s disease.. J. Int. Neuropsychol. Soc..

[29] Cummings J., Zhong K. (2015). Trial design innovations: Clinical trials for treatment of neuropsychiatric symptoms in Alzheimer’s disease.. Clin. Pharmacol. Ther..

[30] Nobis L., Husain M. (2018). Apathy in Alzheimer’s disease.. Curr. Opin. Behav. Sci..

[31] Eikelboom W.S.P., Ossenkoppele R., Coesmans M. (2022). Sex differences in neuropsychiatric symptoms in Alzheimer’s disease dementia: A meta-analysis.. Alzheimers Res. Ther..

[32] Akyol M.A.K., Küçükgüçlü Ö., Yener G. (2019). Investigation of factors affecting apathy in three major types of dementia.. Noro Psikiyatri Arsivi.

[33] Tanaka H., Hashimoto M., Fukuhara R. (2015). Relationship between dementia severity and behavioural and psychological symptoms in early‐onset A lzheimer’s disease.. Psychogeriatrics.

[34] Banning L.C.P., Ramakers I.H.G.B., Köhler S. (2020). The association between biomarkers and neuropsychiatric symptoms across the Alzheimer’s disease spectrum.. Am. J. Geriatr. Psychiatry.

[35] Skogseth R., Mulugeta E., Ballard C. (2008). Neuropsychiatric correlates of cerebrospinal fluid biomarkers in Alzheimer’s disease.. Dement. Geriatr. Cogn. Disord..

[36] Banning L.C.P., Ramakers I.H.G.B., Deckers K., Verhey F.R.J., Aalten P. (2019). Apolipoprotein E and affective symptoms in mild cognitive impairment and Alzheimer’s disease dementia: A systematic review and meta-analysis.. Neurosci. Biobehav. Rev..

[37] Guimarães H.C., Caramelli P., Fialho P.P.A., França E.P., Afonso M.P.D., Teixeira A.L. (2013). Serum levels of soluble TNF-α receptors but not BDNF are associated with apathy symptoms in mild Alzheimer’s disease and amnestic mild cognitive impairment.. Dement. Neuropsychol..

[38] Teixeira A.L., Salem H., Martins L.B., Gonzales M.M., Seshadri S., Suchting R. (2022). Factors associated with apathy in Alzheimer’s disease: A cross-sectional analysis of the texas Alzheimer’s research and care consortium (TARCC) study.. J. Alzheimers Dis..

[39] Holmgren S., Hjorth E., Schultzberg M. (2014). Neuropsychiatric symptoms in dementia—A role for neuroinflammation?. Brain Res. Bull..

[40] Ouanes S.R., Rabl M., Clark C. (2022). Persisting neuropsychiatric symptoms, Alzheimer’s disease, and cerebrospinal fluid cortisol and dehydroepiandrosterone sulfate.. Alzheimers Res. Ther..

[41] Azocar I., Rapaport P., Burton A., Meisel G., Orgeta V. (2022). Risk factors for apathy in Alzheimer’s disease: A systematic review of longitudinal evidence.. Ageing Res. Rev..

[42] Bayard S., Jacus J.P., Raffard S., Gely-Nargeot M.C. (2014). Apathy and emotion-based decision-making in amnesic mild cognitive impairment and Alzheimer’s disease.. Behav. Neurol..

[43] Grossi D., Santangelo G., Barbarulo A.M. (2013). Apathy and related executive syndromes in dementia associated with parkinson’s disease and in Alzheimer’s disease.. Behav. Neurol..

[44] Chau S.A., Chung J., Herrmann N., Eizenman M., Lanctôt K.L. (2016). Apathy and attentional biases in Alzheimer’s disease.. J. Alzheimers Dis..

[45] Aalten P., Verhey F.R.J., Boziki M. (2008). Consistency of neuropsychiatric syndromes across dementias: Results from the European Alzheimer disease consortium. Part II.. Dement. Geriatr. Cogn. Disord..

[46] Hamada C., Kawagoe T., Takamura M., Nagai A., Yamaguchi S., Onoda K. (2021). Altered resting-state functional connectivity of the frontal-striatal circuit in elderly with apathy.. PLoS One.

[47] Powers J.P., Massimo L., McMillan C.T. (2014). White matter disease contributes to apathy and disinhibition in behavioral variant frontotemporal dementia.. Cogn. Behav. Neurol..

[48] Breukelaar I.A., Antees C., Grieve S.M. (2017). Cognitive control network anatomy correlates with neurocognitive behavior: A longitudinal study.. Hum. Brain Mapp..

[49] Jenkins L.M., Wang L., Rosen H., Weintraub S. (2022). A transdiagnostic review of neuroimaging studies of apathy and disinhibition in dementia.. Brain Res. Bull..

[50] Badre D., Hoffman J., Cooney J.W., D’Esposito M. (2009). Hierarchical cognitive control deficits following damage to the human frontal lobe.. Nat. Neurosci..

[51] Seeley W.W. (2019). The salience network: A neural system for perceiving and responding to homeostatic demands.. J. Neurosci..

[52] Cooper J.C., Knutson B. (2008). Valence and salience contribute to nucleus accumbens activation.. Neuroimage.

[53] Bouret S., Richmond B.J. (2010). Ventromedial and orbital prefrontal neurons differentially encode internally and externally driven motivational values in monkeys.. J. Neurosci..

[54] Rosen H.J., Hartikainen K.M., Jagust W. (2002). Utility of clinical criteria in differentiating frontotemporal lobar degeneration (FTLD) from AD.. Neurology.

[55] Boone K.B., Miller B.L., Swartz R., Lu P., Lee A. (2003). Relationship between positive and negative symptoms and neuropsychological scores in frontotemporal dementia and Alzheimer’s disease.. J. Int. Neuropsychol. Soc..

[56] Craig A.D. (2009). How do you feel now? The anterior insula and human awareness.. Nat. Rev. Neurosci..

[57] Allman J.M., Tetreault N.A., Hakeem A.Y. (2011). The von economo neurons in the frontoinsular and anterior cingulate cortex.. Ann. N. Y. Acad. Sci..

[58] Menon V. (2011). Large-scale brain networks and psychopathology: A unifying triple network model.. Trends Cogn. Sci..

[59] Cavanna A.E., Cavanna A.E.T., Cavanna A.E.T. (2006). The precuneus: A review of its functional anatomy and behavioural correlates.. Brain Res. Bull..

[60] Amodio D.M., Frith C.D. (2006). Meeting of minds: The medial frontal cortex and social cognition.. Nat. Rev. Neurosci..

[61] Berridge K.C., Robinson T.E. (1998). What is the role of dopamine in reward: Hedonic impact, reward learning, or incentive salience?. Brain Res. Brain Res. Rev..

[62] Chong T.T.J., Husain M. (2016). The role of dopamine in the pathophysiology and treatment of apathy.. Prog Brain Res.

[63] Le Heron C, Apps MAJ, Husain M (2018). The anatomy of apathy: A neurocognitive framework for amotivated behaviour.. Neuropsychologia.

[64] David R., Mulin E., Friedman L. (2012). Decreased daytime motor activity associated with apathy in Alzheimer disease: An actigraphic study.. Am. J. Geriatr. Psychiatry.

[65] Lanctôt K.L., Herrmann N., Black S.E. (2008). Apathy associated with Alzheimer disease: Use of dextroamphetamine challenge.. Am. J. Geriatr. Psychiatry.

[66] Aston-Jones G., Cohen J.D. (2005). An integrative theory of locus coeruleus-norepinephrine function: Adaptive gain and optimal performance.. Annu. Rev. Neurosci..

[67] Aston-Jones G., Waterhouse B. (2016). Locus coeruleus: From global projection system to adaptive regulation of behavior.. Brain Res..

[68] Szot P., Franklin A., Sikkema C., Wilkinson C.W., Raskind M.A. (2012). Sequential loss of LC noradrenergic and dopaminergic neurons results in a correlation of dopaminergic neuronal number to striatal dopamine concentration.. Front. Pharmacol..

[69] Szot P., Knight L., Franklin A. (2012). Lesioning noradrenergic neurons of the locus coeruleus in C57Bl/6 mice with unilateral 6-hydroxydopamine injection, to assess molecular, electrophysiological and biochemical changes in noradrenergic signaling.. Neuroscience.

[70] Zarow C., Lyness S.A., Mortimer J.A., Chui H.C. (2003). Neuronal loss is greater in the locus coeruleus than nucleus basalis and substantia nigra in Alzheimer and Parkinson diseases.. Arch. Neurol..

[71] Rommelfanger K.S., Weinshenker D. (2007). Norepinephrine: The redheaded stepchild of parkinson’s disease.. Biochem. Pharmacol..

[72] Vazey E.M., Aston-Jones G. (2012). The emerging role of norepinephrine in cognitive dysfunctions of parkinson’s disease.. Front. Behav. Neurosci..

[73] Nakaaki S., Murata Y., Sato J. (2008). Association between apathy/depression and executive function in patients with Alzheimer’s disease.. Int. Psychogeriatr..

[74] Esposito F., Rochat L., Van der Linden A.C.J. (2010). Apathy and executive dysfunction in Alzheimer disease.. Alzheimer Dis. Assoc. Disord..

[75] Drijgers R.L., Verhey F.R.J., Leentjens A.F.G., Köhler S., Aalten P. (2011). Neuropsychological correlates of apathy in mild cognitive impairment and Alzheimer’s disease: The role of executive functioning.. Int. Psychogeriatr..

[76] Wong S., Wei G., Husain M. (2023). Altered reward processing underpins emotional apathy in dementia.. Cogn. Affect. Behav. Neurosci..

[77] Jack C.R., Bennett D.A., Blennow K. (2018). NIA‐AA research framework: Toward a biological definition of Alzheimer’s disease.. Alzheimers Dement..

[78] Marshall G.A., Donovan N.J., Lorius N. (2013). Apathy is associated with increased amyloid burden in mild cognitive impairment.. J. Neuropsychiatry Clin. Neurosci..

[79] Kitamura S., Shimada H., Niwa F. (2018). Tau-induced focal neurotoxicity and network disruption related to apathy in Alzheimer’s disease.. J. Neurol. Neurosurg. Psychiatry.

[80] Mori T., Shimada H., Shinotoh H. (2014). Apathy correlates with prefrontal amyloid deposition in Alzheimer’s disease.. J. Neurol. Neurosurg. Psychiatry.

[81] Johansson M., Stomrud E., Lindberg O. (2020). Apathy and anxiety are early markers of Alzheimer’s disease.. Neurobiol. Aging.

[82] Johansson M., Stomrud E., Johansson P.M. (2022). Development of apathy, anxiety, and depression in cognitively unimpaired older adults: Effects of Alzheimer’s disease pathology and cognitive decline.. Biol. Psychiatry.

[83] Kobayashi H., Ohnishi T., Nakagawa R., Yoshizawa K. (2016). The comparative efficacy and safety of cholinesterase inhibitors in patients with mild‐to‐moderate Alzheimer’s disease: A Bayesian network meta‐analysis.. Int. J. Geriatr. Psychiatry.

[84] Rea R., Carotenuto A., Traini E., Fasanaro A.M., Manzo V., Amenta F. (2015). Apathy treatment in Alzheimer’s disease: Interim results of the ASCOMALVA trial.. J. Alzheimers Dis..

[85] Lee C.W., Chen J.Y., Ko C.C. (2022). Efficacy of methylphenidate for the treatment of apathy in patients with Alzheimer’s disease: A systematic review and meta-analysis of randomized controlled studies.. Psychopharmacology (Berl.).

[86] Kishi T., Sakuma K., Iwata N. (2020). Efficacy and safety of psychostimulants for Alzheimer’s disease: A systematic review and meta-analysis.. Pharmacopsychiatry.

[87] Herrmann N., Rothenburg L.S., Black S.E. (2008). Methylphenidate for the treatment of apathy in Alzheimer disease: Prediction of response using dextroamphetamine challenge.. J. Clin. Psychopharmacol..

[88] Rosenberg P.B., Lanctôt K.L., Drye L.T. (2013). Safety and efficacy of methylphenidate for apathy in Alzheimer’s disease: A randomized, placebo-controlled trial.. J. Clin. Psychiatry.

[89] Raglio A., Bellelli G., Traficante D. (2010). Efficacy of music therapy treatment based on cycles of sessions: A randomised controlled trial.. Aging Ment. Health.

[90] Raglio A., Bellelli G., Traficante D. (2008). Efficacy of music therapy in the treatment of behavioral and psychiatric symptoms of dementia.. Alzheimer Dis. Assoc. Disord..

[91] Kolanowski A., Litaker M., Buettner L., Moeller J., Costa P.T. (2011). A randomized clinical trial of theory-based activities for the behavioral symptoms of dementia in nursing home residents.. J. Am. Geriatr. Soc..

[92] Lam L.C.W., Lui V.W.C., Luk D.N.Y. (2010). Effectiveness of an individualized functional training program on affective disturbances and functional skills in mild and moderate dementia: A randomized control trial.. Int. J. Geriatr. Psychiatry.

[93] Theleritis C., Siarkos K., Katirtzoglou E., Politis A. (2017). Pharmacological and nonpharmacological treatment for apathy in Alzheimer disease: A systematic review across modalities.. J. Geriatr. Psychiatry Neurol..

[94] Tappen R.M., Williams C.L. (2009). Therapeutic conversation to improve mood in nursing home residents with Alzheimer’s disease.. Res. Gerontol. Nurs..

[95] Hattori H., Hattori C., Hokao C., Mizushima K., Mase T. (2011). Controlled study on the cognitive and psychological effect of coloring and drawing in mild Alzheimer’s disease patients.. Geriatr. Gerontol. Int..

[96] Van Weert J.C.M., Van Dulmen A.M., Spreeuwenberg P.M.M., Ribbe M.W., Bensing J.M. (2005). Behavioral and mood effects of snoezelen integrated into 24-hour dementia care.. J. Am. Geriatr. Soc..

[97] Savall ASP, Fidelis EM, de Mello JD (2023). Neuroprotective effect of Eugenia uniflora against intranasal MPTP-induced memory impairments in rats: The involvement of pro-BDNF/p75NTR pathway.. Life Sci.

[98] Kosel F., Hartley M.R., Franklin T.B. (2023). Aberrant cortical activity in 5xFAD mice in response to social and non-social olfactory stimuli.. J. Alzheimers Dis..

[99] Kotredes K.P., Pandey R.S., Persohn S. (2024). Characterizing molecular and synaptic signatures in mouse models of late-onset Alzheimer’s disease independent of amyloid and tau pathology.. Alzheimers Dement..

[100] Kupershmidt L., Youdim M. (2023). The neuroprotective activities of the novel multi-target iron-chelators in models of Alzheimer’s disease, amyotrophic lateral sclerosis and aging.. Cells.

[101] Colucci-D’Amato L., Speranza L., Volpicelli F. (2020). Neurotrophic factor BDNF, physiological functions and therapeutic potential in depression, neurodegeneration and brain cancer.. Int. J. Mol. Sci..

[102] Ghosh A. (2021). Role of norepinephrine in olfactory learning: In young age, in adulthood, and in Alzheimer’s disease PhD Thesis, Memorial Unversityo of Newfoundland.

[103] Oliveira P.A. (2021). The impact of sleep-wake interventions on neuropathology and cognition in a mouse model of dementia.. PhD Thesis, University of Surrey.

[104] Reddy A.P., Sawant N., Morton H. (2021). Selective serotonin reuptake inhibitor citalopram ameliorates cognitive decline and protects against amyloid beta-induced mitochondrial dynamics, biogenesis, autophagy, mitophagy and synaptic toxicities in a mouse model of Alzheimer’s disease.. Hum. Mol. Genet..

[105] Dominguez S.Z.N. (2023). The sex-specific effects of stress in Alzheimer’s disease mouse models..

[106] Liu F., Liu Y., Shen X., Du J., Zhang H., Hou X. (2024). Ovariectomy exacerbates the disturbance of excitation- inhibition balance in the brain of APP/PS-1/tau mice.. Front. Mol. Neurosci..

